# Case report: genetic analysis of a child with 18q deletion syndrome and developmental dysplasia of the hip

**DOI:** 10.1186/s12920-022-01345-2

**Published:** 2022-09-19

**Authors:** Shufeng Yu, Caixia Wang, Ke Lei, Xuefei Leng, Lijuan Zhang, Fei Tian, Zhihong Chen

**Affiliations:** 1 Affiliated Hospital of Qingdao University, Qingdao, China; 2 Qingdao University, Qingdao, China

**Keywords:** 18q deletion syndrome, Developmental dysplasia of the hip, *HSPG2*; fever, Next-generation sequencing

## Abstract

**Objective:**

To analyze the genotypes and phenotypes of a child with developmental dysplasia of the hip (DDH), developmental delays, recurrent fever, hypothyroidism and cleft palate.

**Methods:**

G-banding karyotyping analysis and next-generation sequencing (NGS) were performed for the patient. The genotypes of the parents of the patient were verified by copy number variation analysis and Sanger sequencing to determine the source of variations.

**Results:**

The karyotype of the patient was 46, XX. A 10.44 Mb deletion (chr18:67562936-78005270del) at 18q22.2q23 was found by NGS. We identified 2 *HSPG2* mutations (chr1: 22206699, c.2244C > A, exon 17, p.H748Q; chr1: 22157321–22157321, c.11671 + 154insA, intron). One mutation was inherited from the father, and the other was inherited from the mother.

**Conclusion:**

This is the first 18q deletion syndrome case accompanied by DDH. Most phenotypes of this patient, such as developmental delays and cleft palate, may be related to the 18q22.2q23 deletion, but no variants in genes related to DDH were found in this deletion region. DDH may be related to mutations of *HSPG2.*

## Background

18q deletion syndrome is a rare chromosomal disease with approximately 120 reported cases, and commonly, the deletion is located in the terminal region [1]. The locations and breaking points of chromosomal deletions in 18q deletion syndrome are often different. Moreover, the penetrance of genes in each key region is different, so the clinical phenotypes of this disease vary [2]. The fracture points of the syndrome are generally distributed between 18q11 and 18q23 [3], and most of them are terminal deletions. Some common clinical phenotypes have been identified to be related to the missing coding genes in this region; these phenotypes include short stature, developmental delays, congenital heart disease, cleft palate, IgA deficiency, and hearing loss [4]. There are also some phenotypes that may be related to missing genes, but the related regions have not been accurately located. These phenotypes include autoimmune diseases, cryptorchidism, and hypospadias. Moreover, the causes of some phenotypes, such as fever and hypothyroidism, are currently unknown.

Developmental dysplasia of the hip (DDH) is formerly known as congenital dislocation of the hip. Its incidence rate is approximately 1%, and this rate is approximately 6 times higher in females than in males [5]. The manifestations of the disease include hip dislocation, subluxation and acetabular dysplasia [6, 7], and bilateral dysplasia occurs in approximately 35% of cases. The pathogenesis of DDH is currently considered to be related to genetic and acquired factors [8]. The acquired factors are mainly breech production, oligohydramnios and primipara [9]. At present, there is no report of DDH in children with f18q syndrome; this case is the first reported case. To further explore the relationship between the phenotypes and genotypes of this case, we performed G-banding karyotyping analysis and NGS for the patient. Additionally, the genotypes of the parents of the patient were verified and analyzed by CNV analysis and Sanger sequencing.

## Materials and methods

### Clinical report

**Fig. 1:**
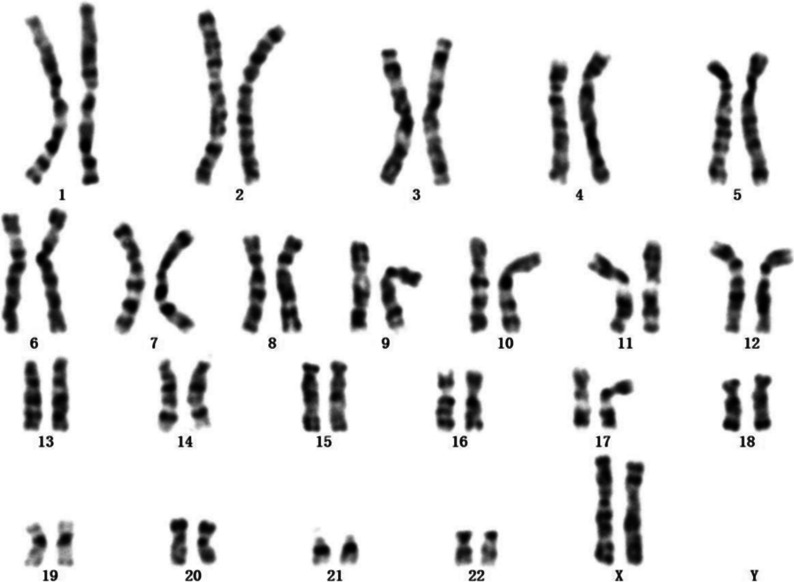
G-banding karyotyping analysis of the child showed 46, XX. No abnormality was found

This patient presented in this case is a female who was 89 days old. She was born to a G1P1 mother by cesarean section due to a breech position at 38 weeks. Her birth weight was 2470 g. She was hospitalized in our hospital because her “body weight increased slowly for 73 days and she had recurrent fever for 11 days.” On the 16th day after birth, the patient was found to have a slow increase in body weight. The thyroid function was considered normal. However, she presented with hypothyroidism, as she had elevated levels of FT3, FT4, and TSH. FT3 was 3.81 pmol/l (normal level: 3.3–8.95 pmol/l), FT4 was 11.57 pmol/l (normal level: 11.9–25.6 pmol/l), and TSH was 82.35 µIU/ml (normal level: 0.73–8.35 µIU/ml). Thyroid ultrasound showed increased thyroid blood flow. Left thyroxine (Euthyrox, 4 µg/kg, Qd) was administered orally. On the 56th day after birth, the patient was diagnosed with bilateral dislocation of the hip (DDH) and fixed with a DDII sling. On the 78th day after birth (11 days before admission), she began to have recurrent fever without coughing, vomiting, or diarrhea. Her parents were not in a consanguineous marriage. Her mother was in good health but suffered from cleft lip. Her father was in good health. Physical examination revealed a temperature of 38.3 °C, a pulse of 145 beats/min, a resting heart rate of 60 beats/min, a weight of 3 kg, a body length of 54 cm, and a head circumference of 38 cm. The patient was in poor spirit. The skin was scattered with patterns. The subcutaneous fat was thin and had poor elasticity. Cleft palate, strabismus, hypotonia were observed. The lower limb was fixed by a DDII sling. Joint ultrasound showed bilateral hip dislocation. Cardiac ultrasound showed patent foramen ovale and tricuspid regurgitation. Brain MRI showed encephalomalacia in the right basal ganglia. Internal auditory canal MRI showed bilateral large vestibular aqueduct dilation, and the brainstem auditory evoked potential examination showed that both ears failed. Chest CT showed a small amount of inflammatory changes in the lungs. The serum IgA was 0.09 g/l (normal range: 0.03–0.78 g/l), IgG was 4.95 g/l (normal range: 1.8–8.0 g/l), IgM was 0.4 g/l (normal range: 0.4–2.3 g/L), and IgE was < 15 IU/ml (normal range: 0–15 IU/ml). Routine blood examination indicated moderate anemia (Hemoglobin was 8.4 g/dl). The thyroid function, CRP, PCT, ESR, routine urine, routine stool, and liver and kidney function tests and the blood gas analysis were generally normal. There was no bacterial growth in multiple blood bacterial cultures, no abnormality in peripheral blood smears, no abnormality in 3-h video EEG, and no abnormality in the G-banding karyotyping analysis (Fig. 1).

After the patient was admitted, we gave her L-thyroxine (Euthyrox, 4 µg/kg/d, Qd, 7 days) by oral administration and piperacillin sodium and tazobactam sodium (100 mg/kg/d, Tid, 7 days) by intravenous administration. The patient’s spirit improved slightly, and her thyroid function was normal. However, she still had a fever. The temperature fluctuated between 37.8 and 39.0 °C. Her skin was dry and she was not sweating. The effect of physical cooling and antipyretics was poor. Due to the patient’s clinical manifestations, we highly suspected that the patient had hereditary diseases. To clarify the diagnosis, we obtained the patient’s blood samples for NGS after obtaining the consent of the patient’s parents.

### Next-generation sequencing (NGS)

Genomic DNA was extracted from the peripheral blood of the patient and her parents, and the whole genome library was prepared by the Beijing MyGenostics company. The gene exon region and its upstream and downstream 50 bp region of the patient and her parents were sequenced by Illumina HiSeq X ten high-throughput sequencing platform, DNBSEQ (DNBSEQ-T7) sequencer, with a read length of 150 bp, sequencing mean depth of 100X.

### Whole genome sequencing and CNV analysis

The data were filtered and compared by the cutadapt (1.16), BWA (0.7.12), and GATK (4.0.8.1) MarkDuplicates software by Beijing MyGenostics Company after sequencing. The CNV was calculated mainly according to the read depth method. All suspicious deleted or repeated regions that were obtained were compared with OMIM, genereviews, decipher, ClinVar, DGV and other databases to obtain the phenotypic information related to the chromosomal regions.

## Results

### NGS results

**Fig. 2:**
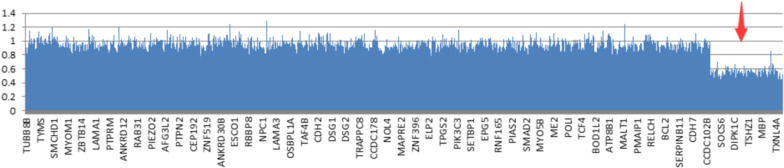
NGS of the Child showed a 10.44 Mb copy number loss at 18q22.2q23

NGS showed that the long arm of chromosome 18 had an approximately 10.44 Mb copy number loss at 18q22.2q23 (67562936-78005270del) (Fig. 2). This region involves the TSHZ1, CTDP1, RBFA, ZNF516, *PARD6G*, MBP, TIMM21, CNDP1, FBXO15, NETO1, KCNG2, ZNF407, NFATC1, ZADH2, TXNL4A, CYB5A, ATP9B, SMIM21, ZNF236, CBLN2, C18orf63, GALR1, SALL3, SLC66A2, CNDP2, DIPK1C, HSBP1L1, ADNP2, RTTN, CD226, and SOCS6 genes. Moreover, we found 108,522 variants, including *HSPG2* mutations (chr1:22206699, c.2244C > A, exon 17, p.H748Q; chr1:22157321–22157321, c.11671 + 154insA, intron) (Table 1), which may be related to DDH.

**Table 1:** *HSPG2* sequencing results

Gene	Chromosome position	Ref_Transcript	Nucleotide/Amino acid Changes	Gene_Type	Allele frequency	Pathogenicity analysis
*HSPG2*	chr1:22206699	NM_005529	c.2244C > A(p.H748Q)	het	0.0002	Uncertain
*HSPG2*	chr1:22157321–22157321	–	c.11671 + 154insA	het	–	Uncertain

### CNV verification results of parents

**Fig. 3:**
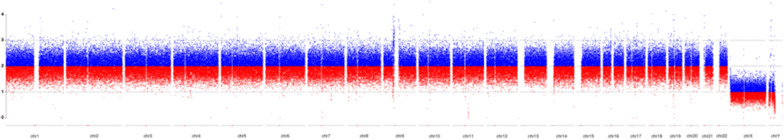
No 18q22.2q23 deletion was found by CNV analysis and verification of the father

**Fig. 4:**
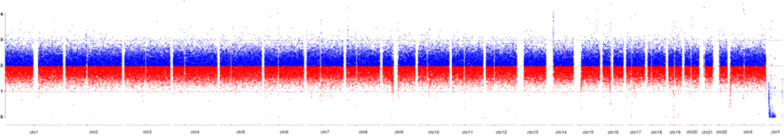
No 18q22.2q23 deletion was found by CNV analysis and verification of the mother

No 18q22.2q23 deletion was found in the CNV verification for the parents (Figs. 3 and 4). The 18q deletion in this patient is a new variation.

### Sanger sequencing of HSPG2 for the patient and her parents

**Fig. 5:**
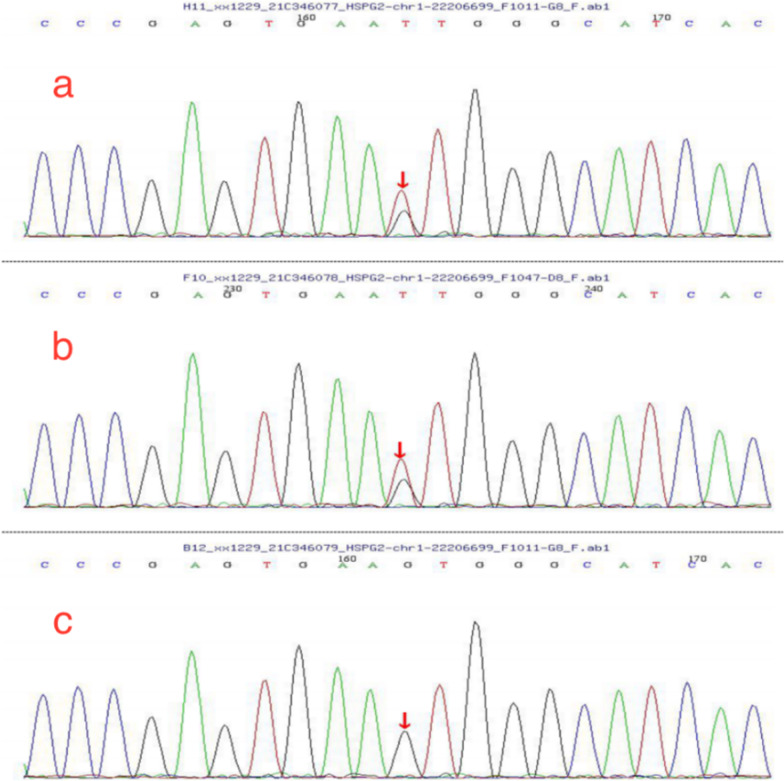
**a** Mutation information *HSPG2* (chr1: 22206699, c.2244c > A, exon 17, p.H748Q) in child; **b** the variation came from the father; **c** the mother had no variation in the site

**Fig. 6:**
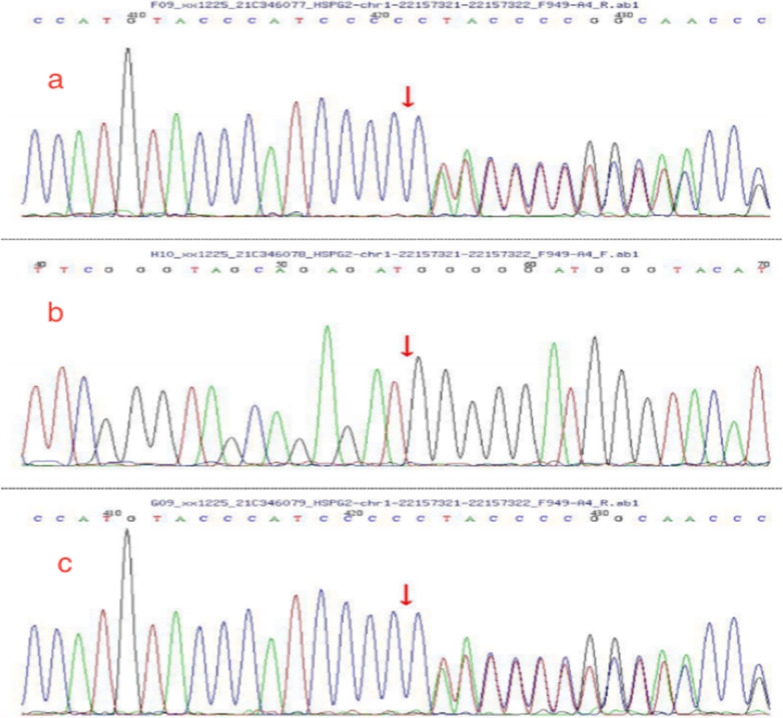
**a** Mutation information *HSPG2* (chr1:22157321–22157321, c.11671 + 154insA, Intron) in child; **b** the father had no variation in the site; **c** the variation came from the mother

Sanger sequencing showed that the variation in the exon site was inherited from the father (Fig. 5), and the variation in the intron site was inherited from the mother (Fig. 6).

***Summary of deletion regions and phenotypes in some reported cases of 18q deletion syndrome*** (Table 2).

**Table 2:** Summary of deletion regions and phenotypes in some reported cases of 18q deletion syndrome

	Chromosome banding	Critical region borders	Size of region (Mb)	Clinical findings
Cody et al. (5 cases) [3]	18q11.2-q21.1	–	19.5	Cryptorchidism, Hypotonia,seizures, vision, hearing loss, Recurrentotitis, Recurrent URI, GU abnormalities, Other MRI findings, developmental delays, hypothyroidism
	18q12.3-q21.1	–	7.5	Hutch diverticuli, hypotonia, hearing loss, recurrent otitis, GU abnormalities, delayed myelination, other MRI findings, developmental delays
	18q12.3-q21.1	–	5	Hypotonia, seizures, developmental delays
	18q12.3-q21.1	–	5	Hypotonia, vision, developmental delays
	18q12.3-q21.1	–	5	Hypotonia, developmental delays
Margarit,E et al. (2 cases) [1]	18q23	71236891–76093303	4.8	GH insuffificiency, dysmyelination, Small adenohypophysis, Renal hypoplasia, Umbilical hernia, Reduced hearing, Smooth philtrum, Thin upper lip, Prognathism, Joint laxity, Neonatal hypoglycemia, developmental delays
Cody et al. (16 cases) [10]	18q22.1	59807588–61568468	17.6	GH insuffificiency, dysmyelination, developmental delays, hearing loss, Foot anomalies, atretic, stenotic ear canals, hypospadias, Tapered fingers, Flat mid-face, proximally placed thumbs, congenital heart abnormalities, Autoimmune disorders (Myalgia, Arthritis, Hypothyroidism), palatal abnormalities, Neonatal complications (Jaundice, Apnea, Respiratory difficulties, Meconium Aspiration), Gastroesophageal reflux
Feng et al. (8 cases) [11]	18q21.31-q23	55040745–78014123	22.973	Cleft palate
	18q21.32-q23	56288429–78013728	21.725	Developmental delays, hearing loss, Atrial septal defect, Cryptorchidism
	18q21.32-q23	56817426–78014123	21.196	Developmental delays, Cleft palate, Atrial septal defect, Strephenopodia
	18q21.33-q23	59581097–78013728	18.432	Flatfoot
	18q21.33-q23	60090078–780013728	17.923	Developmental delays, Cleft palate, Ventricular septal defect, Pulmonary valve stenosis
	18q21.33-q23	61221941–78013728	16.791	Developmental delays
	18q22.1-q23	61985155–78013728	16.028	Developmental delays
	18q22.3-q23	71400740–78013728	6.612	Hearing loss, Pulmonary valve stenosis
Shi et al. [4]	18q22.2-q23	68158880–78014123	9.855	Developmental delays, Hypotonia, Hypothyroidism, Recurrent fever, Seizures, Other MRI findings (abnormal cerebral white matter development, Dysplasia of corpus callosum), Polydipsia, Polyuria
Present study	18q22.2-q23	67562936–78005270	10.44	Developmental delays, Hypotonia, Hearing loss, cleft palate, Other MRI findings (abnormal cerebral white matter development), Vestibular dysplasia, Strabismus, Hypothyroidism, DDH, Recurrent fever

## Discussion

Since the first case of 18q deletion syndrome was reported in 1964 [12], many scholars have tried to study the relationship between phenotype and deletion region or genotype. After years of research, it has been found that some common clinical phenotypes are related to the missing coding genes in those regions. For example, the regions related to short stature and growth hormone deficiency are 18q12.1q12.3, 18q21.1q21.33, and 18q22.3q23, and the possible main pathogenic gene is *GALR1* [1, 13–15]. The region related to developmental delays and abnormal cerebral white matter development is 18q22.3q23, and the possible related pathogenic genes are *ZNF236*, *ZNF516*, *MBP*, *GALR1* and *lOC284276* [2, 16]. The region related to congenital heart disease is 18q22.3q23, and the possible main pathogenic gene is *NFATC1* [17]. The region related to cleft lip and cleft palate is 18q22.3q23, and the possible related pathogenic genes are *SALL3* and *TSHZ1* [18]. The region related to IgA deficiency and renal dysplasia is 18q22.3q23, and the gene related to hearing impairment and abnormal ear development is *ZNF407* [1, 4].

The patient in this case report had many phenotypes, including developmental delays, cleft palate, hypotonia, abnormal cerebral white matter development, hearing loss, vestibular dysplasia, strabismus, hypothyroidism, DDH and recurrent fever. The NGS results revealed that there was a 10.44 Mb deletion at 18q22.2q23. This deletion region contains genes related to developmental delays, short stature, cleft palate, hypotonia, abnormal cerebral white matter development, and hearing impairment. There was a previous case report of a patient with the same missing region as this case, and the phenotypes of this patient included developmental delays, hypotonia, hypothyroidism, recurrent fever, seizures, abnormal cerebral white matter development, dysplasia of corpus callosum, polydipsia, and polyuria [4]. What they had in common was that the deletion region was 18q22.2q23, and both patients presented with developmental delays, hypotonia, hypothyroidism, recurrent fever, and abnormal cerebral white matter development. The differences were that the starting point of the deletion region in our case was 67,562,936, and the end point was 78,005,270. Our case also presented with a cleft palate, hearing loss, vestibular dysplasia, strabismus and DDH. Due to the presentation of these phenotypes combined with the analysis of previously reported cases, we highly suspect that most of the phenotypes in this patient were caused by the 18q22.2q23 deletion. The mother of the patient also had a cleft palate. According to CNV verification, her mother did not have 18q22.2q23 deletion. However, cleft palate is a kind of polygenic genetic disease, and its occurrence is related to many factors. According to the results, cleft palate of the mother may have nothing to do with 18q deletion syndrome, there may be some other chromosomal or genetic abnormalities related to this phenotype.

We did not find any mutations in genes that were related to the recurrent fever phenotype in this patient. Some researchers believe that recurrent fever may be related to the lack of IgA [4], but this child had no symptoms of infection, no growth of bacteria in the blood, normal concentrations of IgA and other immunoglobulins, and ineffective anti-infection treatment. Thus, we inferred that the fever was not associated with IgA deficiency or immune deficiency combined with infection in this case. The patient also had symptoms of low sweat secretion. Recurrent fever may be related to diseases that could cause this symptom, such as abnormal development of the cerebral cortex. Additionally, no pathogenic genes in the deletion region of this patient were found to be directly related to hypothyroidism. However, we found that the *CD226* coding gene was in the deletion region, which encodes a glycoprotein that is expressed on the surface of NK cells, platelets, monocytes and T-cell subsets. This type of glycoprotein mediates the adhesion of platelets and megakaryocytes to vascular endothelial cells. It also plays a role in megakaryocyte maturation [19]. Diseases related to *CD226* mutations include a variety of autoimmune endocrine disorders, such as autoimmune thyroiditis (AITD) [20]. However, the patient was too young to accurately assess autoimmune function. Whether this phenotype is related to this gene deletion needs further study in more cases. Some researchers consider that hypothyroidism is related to abnormal brain development or the thyroid itself [4]; in this case, we cannot exclude the possibility of temporary hypothyroidism.

The patient in this study also had a phenotype that had not been reported in patients with 18q deletion syndrome. The pathogenesis of DDH may be congenital or acquired. Acquired factors mainly include breech delivery. This patient was breech delivered, but she was born by cesarean section without birth injury factors. Therefore, DDH in this case was unlikely to be related to breech delivery. The underlying genetic mechanisms of DDH are not clear at present. The possible genetic mode may be autosomal dominant inheritance with incomplete penetrance, which may be related to variants of *CX3CR1*, *UFSP2*, *HSPG2* and *ATP2B4* [6, 21–23]. NGS analysis indicated that there was a heterozygous variant at the exon site and intron site of *HSPG2* in this patient. The variant at the exon site, c.2244c > A, p.h748q, was a novel mutation from the father who had no clinical manifestation of DDH. The variation at the intron site, c.11671 + 154insa, was located in the deep intron region and was unlikely pathogenic. *HSPG2* encodes for perlecan, a large proteoglycan that plays an important role in cartilage formation, cell adhesion, and basement membrane stability. The diseases related to *HSPG2* mutations mainly include dyssegmental dysplasia Silverman-Handmaker type (DDHS) and Schwartz Jampel syndrome type 1, both of which are autosomal recessive diseases [24]. The clinical features of DDHS include facial dysmorphisms, GU abnormalities, severe short stature, decreased joint mobility, cleft palate and club feet [25]. Schwartz-Jampel syndrome type 1 is characterized by permanent myotonic myopathy and skeletal dysplasia, which result in short stature, dystrophy of epiphyseal cartilages, joint contractures, blepharophimosis, unusual pinnae, myopia and pigeon breasts [26, 27]. The clinical phenotypes of this case were quite different from those of these two diseases. Based on the genetic mode of inheritance, the possibility of these two diseases will not be considered for the time being. Previously, an experiment on *HSPG2* mutant (C1532Yneo) mice [28] confirmed that *HSPG2* mutant mice demonstrated weight loss and DDH. Considering that the genetic mode of inheritance of this disease may be autosomal dominant with incomplete penetrance, the possibility that the DDH in this case is caused by *HSPG2* mutations has not yet been excluded. But, a replication analysis of 250 sporadic samples with Sanger sequencing by Xu et al. [29] indicated that HSPG2 variation was not associated with the susceptibility to DDH in the Chinese Han population. However, whether this phenotype is related to 18q deletion syndrome or other gene mutations needs to be further studied.

## Conclusion

18q deletion syndrome has many common phenotypes with robust differences in clinical manifestations. Most abnormal phenotypes are caused by the deletion of coding genes in the deletion region, but the mechanisms of a few phenotypes are still unknown. Based on the examinations performed in this case, we speculate that the recurrent fever was not related to IgA deficiency. DDH is a newly reported phenotype in a patient with 18q deletion syndrome, which may be related to *HSPG2* mutations in this case. The relationship between phenotypes and genotypes in 18q deletion syndrome need to be further studied.

## Data Availability

The patient data were provided by the Affiliated Hospital of Qingdao University, and the NGS, CNV verification, and Sanger sequencing data were provided by the Beijing MyGenostics company. All data were true and reliable. All data generated or analysed during this study are included in this published article [and its supplementary information files].The datasets generated and/or analysed during the current study are available in the [GSA for Human] repository, [https://ngdc.cncb.ac.cn, the registration number is 2022BAT1983, the accession number is HRA002510].
